# The immaturity of patient engagement in value-based healthcare—A systematic review

**DOI:** 10.3389/fpubh.2023.1144027

**Published:** 2023-05-11

**Authors:** Michael van der Voorden, Wim S. Sipma, Margriet F. C. de Jong, Arie Franx, Kees C. T. B. Ahaus

**Affiliations:** ^1^Department of Obstetrics and Gynaecology, Erasmus University Medical Centre, Rotterdam, Netherlands; ^2^Department of Health Services Management & Organisation, Erasmus School of Health Policy & Management, Erasmus University Rotterdam, Rotterdam, Netherlands; ^3^Department of Nephrology, University Medical Centre Groningen, Groningen, Netherlands

**Keywords:** value-based healthcare (VBHC), patient engagement, quality improvement, patient perspective, co-design, co-production, communication

## Abstract

**Introduction:**

In recent years, Value-Based Healthcare (VBHC) has been gaining traction, particularly in hospitals. A core VBHC element is patient value, i.e., what matters most to the patient and at what cost can this be delivered. This interpretation of value implies patient engagement in patient–doctor communication. Although patient engagement in direct care in the VBHC setting is well described, patient engagement at the organizational level of improving care has hardly been studied. This systematic review maps current knowledge regarding the intensity and impact of patient engagement in VBHC initiatives. We focus on the organizational level of a continuous patient engagement model.

**Methods:**

We performed a systematic review following PRISMA guidelines using five electronic databases. The search strategy yielded 1,546 records, of which 21 studies were eligible for inclusion. Search terms were VBHC and patient engagement, or similar keywords, and we included only empirical studies in hospitals or transmural settings at the organizational level.

**Results:**

We found that consultation, using either questionnaires or interviews by researchers, is the most common method to involve patients in VBHC. Higher levels of patient engagement, such as advisory roles, co-design, or collaborative teams are rare. We found no examples of the highest level of patient engagement such as patients co-leading care improvement committees.

**Conclusion:**

This study included 21 articles, the majority of which were observational, resulting in a limited quality of evidence. Our review shows that patient engagement at the organizational level in VBHC initiatives still relies on low engagement tools such as questionnaires and interviews. Higher-level engagement tools such as advisory roles and collaborative teams are rarely used. Higher-level engagement offers opportunities to improve healthcare and care pathways through co-design with the people being served. We urge VBHC initiatives to embrace all levels of patient engagement to ensure that patient values find their way to the heart of these initiatives.

## 1. Introduction

The concept of value-based healthcare (VBHC) was introduced in 2006 by Porter and Teisberg (1), as a response to the ever increasing and from a societal point of view unsustainable costs of healthcare, a problem that was especially, but not exclusively, present for decades in the US that had the highest costs of care in the world and one of the lowest health indicators (2). In the second half of the 20^th^ century different strategies were pursued to tackle costs varying from fee-for-service payment systems, negotiating prices by both government and private insurers and the introduction of health maintenance organizations (HMOs) for employees. The strategies resulted in a variety of external accountability tools, physicians who feel over controlled and consumer groups (patients) who feel helpless (3). The irony of these approaches was that the system was volume driven, with physicians overproducing thus earning more money and insurers tried to cut both prices and volume in order to control costs. Both mechanisms were not in the interest of patients and people with sickness and diseases. Over the decades new insights were developed that resulted in a growing interest in the concept of value in healthcare. Rather than just focusing on output or lowering costs as isolated management tools, healthcare providers should focus on creating value for patients. Porter and Teisberg introduced the definition of value of any healthcare service as the outcome relative to all the costs incurred to achieve that outcome. They argued that, by following this path, a patient-centered, high quality and affordable healthcare delivery system could be realized. In Europe, EXPH on behalf of the European Commission has defined value broader and introduced four distinct elements of VBHC: personal value (to the patient), technical value (technical achievement), allocative value (distribution of resources), and societal value (contribution to social participation) (4). In this review we limit ourselves to the more narrow definition of VBHC as introduced by Porter and Teisberg (1).

Value for patients is one of the key elements of value-based healthcare (5, 6). To create patient value, in addition to good medical practice, a clear understanding is needed of which outcomes matter most to patients (5, 7). To this end, the use of patient-centered sets of outcome standards is promoted by the International Consortium for Health Outcomes Measurement (ICHOM). Patient Reported Outcome Measures (PROMs) are increasingly used in the consultation room to measure patient-valued outcomes of clinical practice (7–10). In essence, PROMs are questionnaires on a range of health and quality-of-life related issues that are reported by patients themselves and discussed with their doctor.

A second key element of VBHC is the focus on the full cycle of care and the introduction of integrated practice units (IPUs) where care is organized around the needs of patients alongside specialized medical interventions (1, 11). To optimize, from a patient's perspective, the full cycle of care, involving patients in designing patient-centered care pathways can be helpful (12–14). The possibilities and constraints of involving patients in improving health services has been widely studied, including topics such as quality improvement, patient safety, protocol adherence, patient satisfaction, service innovation, and the effectiveness of patient involvement (15–20).

Three frameworks of patient involvement are frequently used (12): Arnstein's (21) ladder of participation, Bate and Robert's (22) continuum of patient involvement and Carman et al.'s (23) continuum of patient engagement. All these frameworks have different angles: Arnstein's (24), a model from the 1960s, focuses on power distribution between actors such as patients and doctors and ignores the value of knowledge diversity. Bate and Robert present a continuum with the most advanced form being experience-based co-design (EBCD) of a care pathway (22). Carman et al. (23) provide a descriptive framework involving three different levels, each along a continuum of patient engagement: the direct care level, the organizational level, and the policy level. On each level, they define a continuum of engagement ranging from consultation through involvement to partnership and shared leadership.

The aim of our study is to present an overview of empirical findings regarding patient engagement in a VBHC context on the organizational level of hospitals. We have chosen to use Carman et al. (23) framework for patient engagement since this makes an explicit distinction between the direct care, the organizational, and the policy levels. The direct-care level is well described in the current VBHC literature and includes outcome measurements, shared decision-making, and costs (8, 21, 22). The policy level concerns societal issues related to healthcare and is only loosely linked to day-to-day clinical practice. Consequently, this study focuses on the organizational level, covering the hospital unit through to designing the full cycle of care, which is hardly described from the perspective of patient engagement (3, 23).

## 2. Methods

This systematic review is conducted and reported following the protocol of Prisma Guidelines for systematic reviews (25). Details are provided in Supplementary material 1. In addition, the authors are trained researchers and the team is highly experienced in conducting systematic reviews. The review was not registered.

### 2.1. Search strategy

The search strategy was developed in collaboration with an expert librarian from the Erasmus University Medical Centre, Rotterdam, Netherlands. Five databases were searched on 14-01-2022: Embase, Medline ALL, Web of Science Core Collection, Cochrane Central Register of Controlled Trials, and Google Scholar. The search strategy followed PICO to formulate the definitions of the research question. (1) P (patient/ population), patients in a hospital or transmural setting, (2) I (intervention), value-based healthcare, (3) O (outcomes), patient engagement on an organizational level of hospitals. The C (comparator) is not applicable in this study. The search strategy consisted of the two major elements of this systematic review, patient participation and value-based healthcare, plus their plural forms. Supplementary material 2 provides the full search string. Duplications of any articles were excluded. References were cross-checked and added if not already included. Seven clearly relevant papers were identified in advance of this to check that the search strategy correctly retrieved them.

### 2.2. Selection process

In advance of the full selection process, five articles were independently screened by two researchers (MV&WS) by title and abstract to check for agreement on inclusion and exclusion criteria. The results were discussed by the two researchers and the results of the screening by these two researchers were fully agreed by both. We made the choice to use Rayyan as a tool to streamline the process. Herewith, both researchers independently screened the titles and abstracts of the papers identified in the search. After both researchers (MV & WS) had screened these articles, they were uploaded in one overview. There was discussion about articles when there was a discrepancy between the two researchers. Consensus was found between the two researchers and these articles proceeded to full text screening. The reasons for excluding a research paper during the next stage, full text screening, were recorded and inconsistent screening outcomes were discussed by the two researchers. Six articles where there was no consensus were reviewed by two other researchers (AF & KA) with four being included and two rejected.

### 2.3. Eligibility criteria

The eligibility criteria were applied in two phases: title and abstract screening and full text screening. In the first phase (title and abstract screening), the criteria for excluding the studies were “mentioned value-based healthcare as a research topic but no patient engagement or vice versa”, “setting other than a hospital environment or transmural”, “research papers prior to 2006”, “not written in English”, “not peer reviewed”, “not empirical research”, and “conference paper”. We did not include papers published prior to 2006 because Porter and Teisberg (1) introduced the concept of value-based healthcare in 2006. In the second phase (full text screening), patient engagement and value-based healthcare were further explored. The primary outcomes were the level of patient engagement on an organizational level and the integration of the VBHC elements in practice. Patient engagement was defined as active participation by patients in the study described in the research paper. Based on the framework by Carman et al. we investigated the level of patient engagement from an organizational unit (meso-level) perspective. Carman et al.'s model presents a continuum of engagement, whereby consultation, involvement and partnership, and shared leadership are used to define the level of patient engagement from low to high (23). The criteria for excluding the initially identified studies were “direct care (micro-level) in a hospital setting”, “macro-level care in a hospital setting”. This research was aimed at synthesizing the information from the studies that developed new knowledge about patient engagement from an organizational-unit perspective in a value-based healthcare context which was the focus of our study. An integrative approach has been chosen in this systematic review, since the ways in which patients participate in both qualitative and quantitative data can be investigated.

### 2.4. Data extraction and analysis

Data extraction consisted of three steps of thematic analysis and these were carried out independently by two researchers (MV & WS). Atlas.ti, version 22 was used to facilitate this process. First, the generic characteristics of a study were examined in terms of authors' names, the year of publication, country, medical specialties involved, study design, and number of patients involved. Second, the context of the study in terms of the field of value-based healthcare was examined. We looked for the presence, or absence, of the elements of value-based healthcare proposed by Porter and Teisberg (1): value, outcomes, and costs, and how these were used in the practical design of the study. Finally, to examine the context of patient engagement, we inductively analyzed how patients were involved, what level of patient engagement was apparent based on the model of Carman et al. which patient engagement outcomes were reported and to what extent the participation of patients contributed to the results of the study.

Due to the focus of the research question, a narrative approach has been chosen for displaying and presenting the data in tables. As a result, a meta-analysis was not undertaken and the results were analyzed descriptively and thematic. This was necessary given the studies' heterogeneity for study designs, participants, objectives and results.

### 2.5. Quality assessment

The mixed methods appraisal tool (MMAT) (26) was used to assess the quality and risk of bias in the 21 studies included. The MMAT was developed for systematic reviews that combine qualitative, quantitative, and/or mixed studies (27, 28). Moreover, the MMAT was developed for the appraisal stage of systematic reviews and facilitates the appraisal of empirical studies including observational studies. MMAT facilitates the appraisal of five research categories: qualitative research, randomized controlled trials, non-randomized studies, quantitative descriptive studies, and mixed methods studies. Following the quality criteria as described in the MMAT user guide, two researchers (MV & WS) have both independently of each other assessed each study and after discussion, the scores were decided together. The qualitative (*n* = 5), quantitative (*n* = 9), and mixed-methods (*n* = 7) studies were subjected to their own screening categorization that involves a set of five unique criteria. For each criteria, a “yes” response was scored “1” and a “no” or “can't tell” scored “0”. An overall score of “5” means that all the quality criteria are met; a score of “0” that none of the quality criteria are met (26). We converted this to the score “5” is high, score “4” and “3” is medium, and “2”, “1” and “0” is low.

## 3. Results

**Figure 1:**
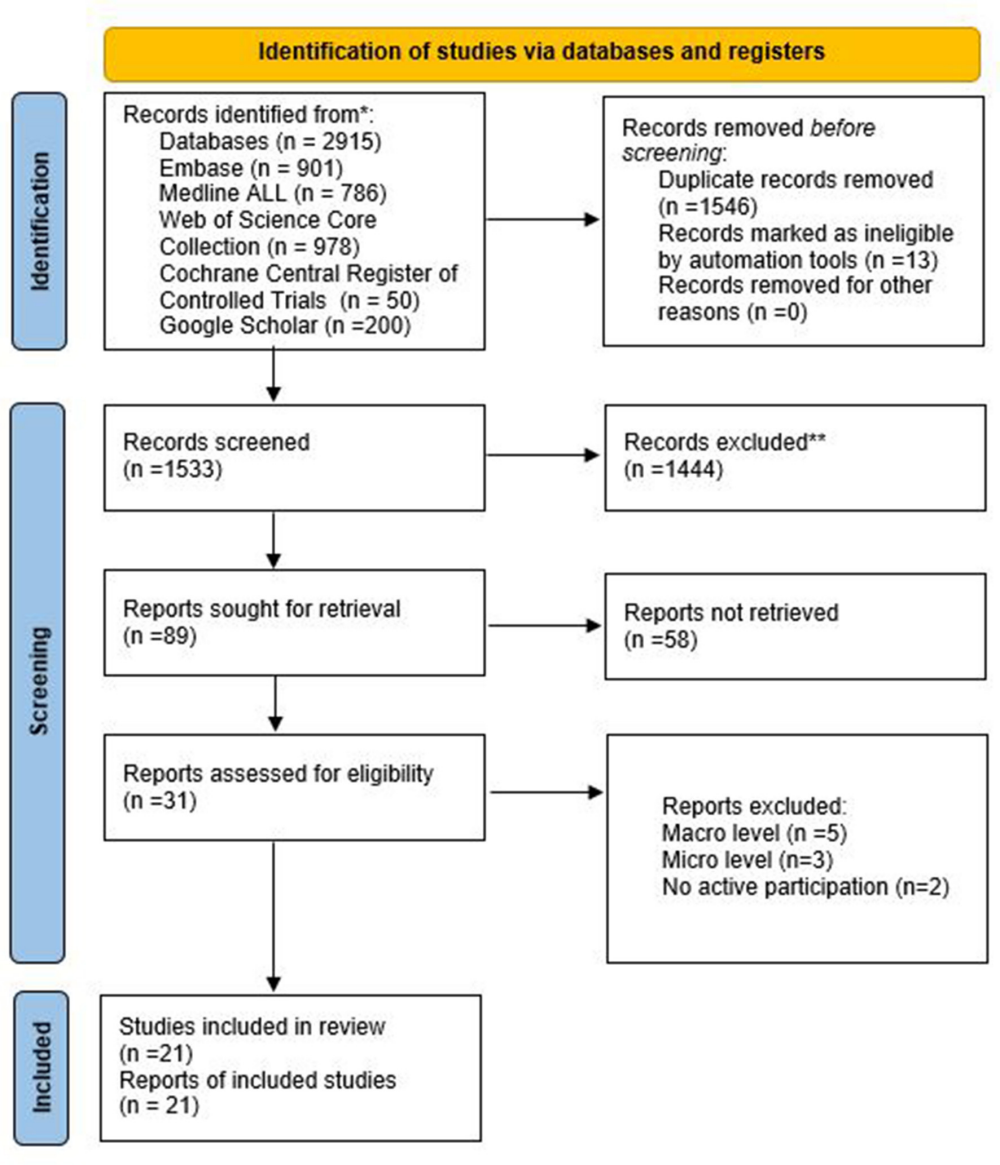
PRISMA flow diagram.

The search strategy yielded a total of 2,915 articles across the five databases. A total of 1,533 articles remained after removing duplicates. A total of 21 articles remained after the title and abstract, followed by full text, screening. Further details can be found in Figure 1 above.

A schematic representation of all results of the 21 studies is provided in Table 1 below.

**Table 1:** General characteristics and relevant elements of the studies.

**References**	**Country**	**Specialties involved**	**Study design**	**Number of patients involved**	**Value-based healthcare elements**	**Objective**	**Level of participation**	**Results of patient participation**	**Illustrative quote**
Anderson et al. (29)	United States	Not mentioned	Qualitative	23	Value	To create an understanding of to what extent seriously ill patients value a cardiopulmonary resuscitation (CPR) discussion with their doctor.	Involvement	A strong doctor-patient relationship was an essential context for CPR discussions. Participants also valued relationships with hospital doctors. In total of 50% reported no preference between the videos; 35% preferred the information-focused one, and 15% the value-based video.	“*After viewing both videos, participants were asked which model they would prefer for discussing CPR with a hospital doctor”*
Hennink et al. (30)	The Netherlands	Gastroenterology Clinical genetics	Quantitative	64	Value, outcome, and costs	Lynch Syndrome patients evaluated the care delivered to these patients in their department and formulated outcome measures relevant to patient value.	Involvement	The relevance of all six outcomes was confirmed by the patients in the survey and mean scores varied from 7.2 to 9.9.	“*These patients were invited to participate in this survey and to complete a questionnaire that assessed the importance of the outcomes (on a scale 1–10) in the cycle of care identified by the specialists”*
Van Citters et al. (31)	United States	Orthopedics Anesthesiology	Mixed methods	2	Value	1: To develop a generalizable care pathway using inputs from clinical, academic, and patient stakeholders. 2: Identify system and patient-level processes to provide safe, effective, efficient, and patient-centered care.	Consultation involvement	Study used different stakeholder categories to develop a generalizable care pathway that outlines 40 processes to improve care, 37 techniques to avoid waste, and 55 techniques to improve communication.	“*Patient-level discussions were designed to validate concepts identified by care teams and included pleasing and disappointing features of care; factors that are contributed to safety, efficiency, or patient and family experience; and advice for providers”*
Kaplan et al. (32)	United States	Urology	Qualitative	7	Value, outcome, and costs	To implement patient ethnography to support the quality improvement infrastructure and improve patient centeredness.	Consultation	Themes emerged from the interviews that were considered of low value to the patient, they had identified five improvements targeted at the low-value themes. Three of these had been implemented.	“*These discussions sought to understand patient perspective, context and the care experience surrounding their treatment”*
Li et al. (33)	United States	Gastroenterology	Quantitative	53	Value, outcome, and costs	To test the hypothesis that telemedicine in the form of telecare will increase value while achieving high satisfaction for patients with inflammatory bowel disease.	Consultation involvement	The telemedicine clinic enabled patients to save, on average, $62 in out-of-pocket costs. In 77% of the patients continued to use telemedicine as their preferred follow-up method.	“*After the visit, the patients fills out a postvisit survey that included questions about the patient's experience of the visit, time and money saved by not driving the appointment, and preference for further visits”*
Bernstein et al. (34)	United States	Orthopedics	Quantitative	185	Value, outcome, and costs	To determine if PROMIS, used as a part of routine orthopedic clinical care, is associated with improved patient experience.	Consultation	Patients who used PROMIS were 89% more likely to feel that the provider spent enough time with them, 81% more likely to recommend this provider office to another patient, and rated the provided significantly higher on a scale from 0 to 10.	“*Only the subset of CGCAHPS questions directly related to patient experience and satisfaction were included”*
Coppess et al. (35)	United States	Pediatrics	Quantitative	56	Value, outcome, and costs	To evaluate whether an OPPS/cost method can be used for value-based evaluation of healthcare delivery with patient experience as an element of it.	Consultation	A 1.7% reduction in costs, improvement in objective and subjective outcomes of 47.4 and 7.3% respectively, and stable patient experience was seen with the clinic location change.	“*Patient/family experience is a measure of satisfaction with the guardian/provider interaction. The survey is performed via email and phone within 3 days after a patient's visit and scored utilizing an structured query language script as standard practice for the institution independent of this study”*
Eppler et al. (36)	United States	Hand surgery	Qualitative	99	Value, outcome, and costs	To develop a better understanding of the surgery and recovery experience of hand surgery patients, specifically focusing on knowledge gaps, experience, and the surgical process.	Consultation involvement	Four themes were developed from the thematic analysis: (1) being prepared and informed for the process of surgery, (2) regaining hand function without pain or complication, (3) patients and caregivers negotiating the physical and psychological challenges of recovery, and (4) financial and logistical burdens of undergoing hand surgery.	“*The questionnaire was composed of 8 open-ended questions, asking about various aspects of their treatment, and recovery including patient education, challenges, preparation, and success”*
Rosseel et al. (37)	Denmark	Cardiology	Quantitative	637	Value, outcome	To deliver data on patients' perceived values and health-related quality of life following surgical aortic valve replacement (SAVR) and transcatheter aortic valve replacement (TAVR) in a real-world, all-comers patient population.	Consultation	Both physical (42 vs. 11%) and mental (30 vs. 11%) impacts of the intervention and the recovery period were experienced as more stressful by SAVR as compared to TAVR patients. In both groups, 10% of the patients reported no change in health-related quality of life (HR-QoL), whereas HR-QoL improved in 76 vs. 83% and worsened in 14 vs. 7% of the SAVR and TAVR populations, respectively.	“*The questionnaires in this study were specifically designed to capture patients and informal caregivers' perioperative experience as well as the patients' HR-QoL before and after aortic valve replacement”*
Wickramasinghe et al. (38)	Australia	Obstetrics Gynecology	Mixed methods	10	Value	The study was designed to assess patient compliance, satisfaction, level of glycemic control achieved, and healthcare professional satisfaction.	Consultation involvement	From the patient perspective, five *a* priori themes were included, and thematic analysis served to reveal two others. In addition, all patients preferred to have standard care plus the mobile solution rather than only the standard care approach. Many ideas for further enhancement were provided by the patients.	“*The questionnaire at the conclusion of the study was designed to ascertain overall satisfaction with the technology solution and allow for any recommendations moving forward”*
Depla et al. (39)	The Netherlands	Obstetrics Gynecology	Mixed methods	26	Value, outcome	To study the feasibility of using PROMs and PREMs in Dutch perinatal care, addressing both women's and professionals' perspectives.	Consultation Involvement	The majority of women (76%) wanted to discuss their PROM answers with a care professional, and 81% their PREM answers. Most women (86%) preferred to discuss their answers with an obstetric care professional. Over half of the women agreed that PROMs/PREMs supported shared decision-making (58%), ability to raise issues (60%), and the patient-clinician relationship (52%).	“*To evaluate usability and experiences, separate evaluation surveys were composed for both patients and obstetric care professionals, regarding barriers and facilitators to using the PROM/PREM questionnaires in daily practice”*
Dronkers et al. (40)	The Netherlands	Oncology	Mixed methods	166	Value, outcome	To provide an initial evaluation of Healthcare Monitor (HM) after implementation and seek new insights into how patients experience HM.	Consultation involvement	HM users more often experienced that their physician had a complete picture of them and took action in response to their specific complaints.	“*Patients were interviewed on the added value of HM and on how they think of HM in general” “We also asked questions about the length of the consultation and asked patients to rate their subjectively experienced quality of care ranging between 1 and 10”*
Fahner et al. (41)	The Netherlands	Pediatrics	Qualitative	20	Value	To clarify how parents of children with life-limiting conditions contemplate the future and under which conditions parents share these future perspectives with clinicians caring for their child.	Involvement	Four main themes were identified when parents were asked to envision the future of their child. It was seen that: 1) there is a focus on the near future, 2) future perspective are intertwined with present and past experiences, 3) future perspectives range from a disease-related orientation to a value-based orientation, and 4) there is no “sharing without caring”.	“*Several triggers stimulated them to contemplate the future. [..] These questions made parents think about their underlying values and influence of these values on future decision making”*
Goretti et al. (42)	Italy	Bariatric surgery	Mixed methods	2,122	Value, outcome, and costs	To redesign the organizational bariatric pathway by implementing a VBHC strategy to achieve excellent clinical outcomes and improved quality of life without increasing costs.	Consultation involvement	There were three categories of recommendations with a total of seven elements that formed the basis for redesigning the bariatric pathway. The interventions confirm the positive impact of bariatric surgery on clinical outcomes and significant improvements in the quality of life for morbidly obese patients.	“*Patients were interviewed by clinicians to collect their experiences and suggestions to improve their pathway of care”*
Pennucci et al. (43)	Italy	Cardiology	Mixed methods	162	Value, outcome	To evaluate the feasibility of a digital-based continuous collection and reporting of PROMs and PREMs for patients with chronic heart failure.	Consultation	The system has been successfully implemented. Response rates have been consistently above 50%, demonstrating patients' willingness to participate. All the involved stakeholders acknowledged the feasibility of the design.	“*At baseline, patients were asked questions exploring the quality of care before the index hospitalization and during the hospital stay. [..] After 1 month, the questions were related to the experience of care during the hospitalization”*
Van Veghel et al. (44)	The Netherlands	Cardiology	Quantitative	669	Value	To evaluate the effects of a pilot study regarding enhancing regional integration on patient-relevant clinical outcomes and patient satisfaction.	Consultation	The non-significant improvement has, over time, led to significantly better outcomes for patients referred from the study-referring hospital compared to patients referred from other hospitals. The level of satisfaction improved and achieved statistically significant higher scores for various items.	“*On a scale from 1 to 10, patients where asked “To what extent are you satisfied with…”, followed by the specific 28 items. [..] Patients were asked to give an overall grade of the delivered care in both hospitals on a scale from very bad (=1) to excellent (=10)”*
Young et al. (45)	United States	Intensive care Neurosurgery	Quantitative	269	Value, outcome	To evaluate the clinical and financial outcomes, as well as the impact on the patient experience, for patients who participated in the STP and bypassed the intensive care unit (ICU) level of care	Consultation	Admitting selected, but generally otherwise healthy, postoperative craniotomy patients directly from the PACU to the step-down unit, bypassing the ICU, is safe and, as one might expect, can result in cost savings ($422,128) and does not adversely affect the patient (73 vs. 86%) or provider experience (87.5%).	“*Surveys were distributed for patients and nurses to document their satisfaction with the program”*
Ahluwalia et al. (46)	UnitedKingdom	Orthopedics	Quantitative	53	Value, outcome and costs	To assess the safety, efficiency, cost-effectiveness, and differences in clinical and patient outcomes of day surgery unit (DSU) care for ankle fracture treatment	Consultation	The DSU pathway improves the value of healthcare delivery with high patient satisfaction scores when compared to the traditional pathway (7.7 vs. 6.3). The model demonstrates predictably good clinical outcomes (no associated complications) with a financial cost benefit (£2018) over the in-patient admission care model for selected patients.	“*A telephone satisfaction poll was conducted [..] patient satisfaction was graded out of 10, with 10 representing exemplary service continuing down to 0, which represents the worst healthcare experience possible”*
Najafabadi et al. (47)	The Netherlands	Neurosurgery Radiotherapy	Mixed methods	31	Value	To evaluate the structure of current meningioma care and identify issues and potential high-impact improvement initiatives.	Involvement	Following the grounded theory approach, issues were eventually categorized into a thematic framework consisting of the following three themes: (1) availability and provision of information, (2) care and support, and (3) screening for (neurocognitive) rehabilitation. Following up on these issues, 16 solutions were identified during focus groups.	“*Using the thematic framework from step 1, participants were asked to identify issues regarding their meningioma care trajectory, as well as possible solutions for these issues”*
Slejko et al. (48)	United States	Pulmonology	Qualitative	31	Value, outcome, and costs	To elicit from patients with chronic obstructive pulmonary disease their prioritization of an established set of patient-informed value elements.	Involvement	Initially, participant responses informed the selection of eight elements as the key aspects for the Phase 2 language refinement. With feedback from a patient advocate, and additional patient participants, elements were refined, rephrased, or modified, and the list was reduced to six value elements.	“*We developed an instrument that first asked participants about the clarity of an overarching choice task question for the future-stated preference instrument. Next, we asked participants to consider a proposed statement for each of the elements retained after Phase 1, framed as attributes”*
Kasalak et al. (49)	The Netherlands	Radiology	Quantitative	58	Value	To investigate how patients experience a radiologist—patient consultation of imaging findings directly after neck ultrasonography (US), and how much time this consumes.	Consultation	Patients who did not discuss the US results with the radiologist were significantly more worried during the examination (*P =* 0.040) and had significantly higher anxiety levels after completion of the US examination (*P =* 0.027) than patients who discussed the results with the radiologist. The median duration of US examinations that included a radiologist-patient consultation was 7.57 min compared with 7.34 min for those without this consultation.	“*Patients in both randomization arms were asked to fill in a paper-based survey to share their experience with the US examination and their view on the radiologist-patient consultation of US results at the end of the examination”*

### 3.1. General characteristics

The studies were all published between 2013 and 2022. Nine studies were conducted in the United States (29, 31–36, 45, 48), seven studies in The Netherlands (30, 39–41, 44, 47, 49), two in Italy (42, 43), one in Australia (38), one in Denmark (37), and one in the United Kingdom (46). Nine studies had a quantitative design (30, 33–35, 37, 44–46, 49); seven studies a mixed methods design (31, 38–40, 42, 43, 47) and five a qualitative design (29, 32, 36, 41, 48). In total, 4,743 participants were involved in the 21 studies, ranging from 2 patients (31) to 2,122 patients (42). All 21 studies were conducted in hospitals: 14 studies in a single hospital (29, 30, 33–35, 37, 40–43, 46–49), five studies in multiple hospitals (31, 36, 38, 44, 45), one in a single hospital and in patients' homes (32), and one in multiple hospitals and at home (39).

### 3.2. Quality assessment

The quality assessment resulted in classifications of “high” (13 studies), “medium” (8 studies), with none categorized as “low”. Consequently, no studies were excluded on the basis of the MMAT. Overall, the quantitative studies tended to achieve higher quality scores than the mixed methods and qualitative studies. A detailed overview and the explanations of the scores of the quality assessment are provided in Supplementary material 3.

### 3.3. Patient participation in a value-based healthcare context

#### 3.3.1. Value-based healthcare context

Nine studies discussed value, outcome, and costs in relation to each other (30, 32–36, 42, 46, 48) of which six investigated the costs from an organizational perspective (30, 32, 34, 35, 42, 46) and three from a patient perspective (33, 36, 48). Five other studies discussed both value and outcomes but not costs (37, 39, 40, 43, 45), three of which investigated patient-reported outcomes (39, 40, 43) and two studies clinical outcomes (37, 45). Seven studies discussed only value (29, 31, 38, 41, 44, 47, 49). In these articles, the focus on value was not linked to outcomes and costs, but more on patient value in terms of what patients consider important.

### 3.4. Levels of patient engagement

Nine studies indicated only the level of consultation (32, 34, 35, 37, 43–46, 49), and five the level of involvement (29, 30, 41, 47, 48). In addition, seven studies included both these aspects of patient engagement (31, 33, 36, 38–40, 42). None of the studies reported patient engagement at the “partnership and shared leadership” level. A schematic representation is provided in Table 2 below.

**Table 2:** Studies at the different levels of patient engagement.

	**Consultation**	**Consultation and involvement**	**Involvement**	**Partnership and shared leadership**
Direct care (micro-level)				
Organizational design and governance (meso-level)	(32, 34, 35, 37, 43–46, 49)	(31, 33, 36, 38–40, 42)	(29, 30, 41, 47, 48)	
Policymaking (macro-level)				

The findings in the reviewed papers, insofar as they relate to the levels of patient engagement that emerged from the thematic analysis, in terms of level of engagement, type of studies and their modalities, data collection methods, role of patients related to the level of patient engagement, outcomes patient engagement and results of patient engagement reported are included in three different tables. The details for each level are discussed below.

### 3.5. Consultation

For the nine studies (32, 34, 35, 37, 43–46, 49) that were limited to the consultation level of patient engagement (23), we investigated how the level of engagement “consultation” was implemented in practice. Further details can be found in Table 3 below.

**Table 3:** Consultation level of engagement.

**References**	**Level of engagement**	**Type of studies**	**Modalities**	**Data collection methods**	**Role of patients related to the level of engagement**	**Outcomes patient engagement**	**Results of patient engagement reported**
Kaplan et al. (32)	Consultation	Observational	Cohort study	Interviews	Patients were asked in the interviews about their perspective, context and care experience. The central question at the crux of each interview was about what about the care was dissatisfying.	Patient experience Quality of care	Five improvements emerged from the interviews with patients, three of them have been implemented
Bernstein et al. (34)	Consultation	Observational	Cross-control study	Questionnaire/survey	Patients filled out a questionnaire/survey and only the questions directly related to patient experience and patient satisfaction were included.	Patient experience Patient satisfaction	The questionnaire showed significantly higher scores for patients that used PROMIS and the PROMIS has a positive impact on the patient experience
Coppess et al. (35)	Consultation	Observational	Cross-control study	Questionnaire/survey	Patients filled out a questionnaire/survey by phone or e-mail within 3 days after a visit and scored patient satisfaction on scales ranging from 3 to 11.	Patient satisfaction	Results for patient satisfaction are mentioned as part of the OPPS/COST method
Young et al. (45)	Consultation	Observational	Cross-control study	Questionnaire/survey	Patients were given a satisfaction survey to assess their respective impressions of the hospital stay and of the recovery pathway.	Patient satisfaction	The conclusions focused heavily on clinical and financial outcomes, and just looked to check that there were no adverse patient experiences
Van Veghel et al. (44)	Consultation	Observational	Cohort study	Questionnaire/survey	Patients were asked to fill out a questionnaire/survey on patient satisfaction on a scale from 1 (very bad) to 10 (excellent).	Patient satisfaction	The patient satisfaction results were used to further improve care management and promote the quality of outcomes for referred patients
Kasalak et al. (49)	Consultation	Randomized	Prospective randomized study	Questionnaire/survey	Patients were asked to fill out a paper based questionnaire/survey to share their experience and their view on the consultation.	Patient experience Patient perspective	Compared two groups of patients and concluded that the group that underwent neck ultrasonography were generally satisfied
Pennucci et al. (43)	Consultation	Observational	Cohort study	Questionnaire/survey Workshop	Patients were asked to measure disease-specific outcomes and they were asked questions related to patient experience and quality of care	Patient experience Quality of care	The inputs of patients allowed the feasibility of the design to be acknowledged
Rosseel et al. (37)	Consultation	Observational	Cross-control study	Questionnaire/survey	To capture patients perioperative experience as well as the patient's health-related quality of life before and after aortic valve replacements	Patient experience Patient health-related quality of life	The results of patient experiences were used to make a comparison between SAVR and TAVR patients
Ahluwalia et al. (46)	Consultation	Observational	Cohort study	Telephone satisfaction poll	Patients were asked by a telephone satisfaction poll how satisfied they were on a scale of 1 (worst experience) to 10 (exemplary service).	Patient satisfaction	The results showed the benefits, both clinical and financial outcomes, and patient satisfaction was a part of this

#### 3.5.1. Type of studies and their modalities

Eight of nine studies have an observational study design (32, 34, 35, 37, 43–46) of which four are cohort studies (32, 43, 44, 46), and four cross-control studies (34, 35, 37, 45). One study has a randomized study design, which is a prospective randomized study (49).

#### 3.5.2. Data collection methods

Seven of nine studies used either a questionnaire or a survey (34, 35, 37, 43–45, 49). In one study the questionnaire or survey was combined with a workshop (43). One study relied completely on interviews for collecting data (32), and another used a telephone satisfaction poll (46).

#### 3.5.3. Outcomes of patient engagement

Based on the role of patients, one or two outcomes were reported. Five of nine studies reported two outcomes in their article (32, 34, 37, 43, 49) and four studies one outcome (35, 44–46). Five studies reported patient satisfaction as outcome of patient engagement (34, 35, 44–46), five studies patient experience (32, 34, 37, 43, 49), two studies quality of care (32, 43), one study patient perspective (49) and one study patient health- related quality of life.

#### 3.5.4. Results of patient participation reported

Six of nine studies show that the role of patients and their input is substantial used in the results and conclusion of the study (32, 34, 37, 43, 44, 49), two studies show that the results were included as part of more results (35, 46) and one study shows that is used minimally in the results (45). In the studies which the results were included as part of more results, one study focuses either on financial and clinical outcomes (46) and one either on financial outcomes (35). In the study which the results were minimally used focused heavily on financial and clinical outcomes (45).

### 3.6. Consultation and involvement

Seven studies addressed the level of consultation and involvement (31, 33, 36, 38–40, 42). Further details can be found in Table 4 below.

**Table 4:** Consultation and involvement level of engagement.

**References**	**Level of engagement**	**Type of studies**	**Modalities**	**Data collection methods**	**Role of patients related to the level of engagement**	**Outcomes patient engagement**	**Results of patient engagement reported**
Van Citters et al. (31)	Consultation and involvement	Observational	(Multiple) case study	Interviews multi-stakeholder panel	Patients participated in a semi-structured telephone interview and included factors that contributed to safety, efficiency, or patient and family centered care experience (consultation) and validation of concepts and advice for providers in terms of improving care and efficiency (involvement).	Patient experience Validation of concepts Patients' advice for providers	Developed a generalized care pathway with various stakeholders, but patients were just a small part of it (2/48)
Li et al. (33)	Consultation and involvement	Observational	Cohort study	Questionnaire/survey	Patients filled out a pre-visit questionnaire/survey about their current disease state including quality outcome measures. After the visit, the patients filled out a questionnaire/survey with questions related to the patient's experience of the visit, time and money saved by not driving to the appointment (consultation) and preference for further visits (improvement).	Quality outcome measures Patient experience Patients' time and money saved Patient preferences	Based on the patients' answers, their experiences, the time and money saved, and the preferred follow-up method were mentioned
Eppler et al. (36)	Consultation and involvement	Observational	Cross-sectional study	Questionnaire/survey	Patients were asked to fill out an open-ended questionnaire/survey with 8 questions about various aspects of their treatment, and recovery including patient education, challenges, preparation and success (consultation). Patients were asked to respond and gave feedback on the questionnaire (involvement).	Treatment aspects Challenges for recovery Feedback/advice of patients on a questionnaire	The high-quality criteria based on the patients' answers are clearly categorized in four themes
Depla et al. (39)	Consultation and involvement	Observational	Cross-sectional study	Questionnaire/survey Focus group	Patients were asked to fill out a questionnaire/survey at one time-point (T1-T5) for patient experiences and patient preferences (consultation. To evaluate usability and experiences, separate questionnaires/surveys were composed, regarding barriers and facilitators to using the questionnaire/survey in daily practice (involvement).	Patient experience Barriers and facilitators	Both women's and professionals' experiences and barriers and facilitators were noted as equally important
Dronkers et al. (40)	Consultation and involvement	Observational	Cross-control study	Questionnaire/survey Interviews	Patients were asked to fill out a 12-item patient experience questionnaire with a 4 point Likert scale (consultation). Patients were interviewed on the added value of Health Monitor and how they think of HM in general in terms of barriers and facilitators (involvement).	Patient experience Barriers and facilitators Added value	The experiences of patients that used and did not use the healthcare monitor were compared and the barriers and facilitators that patients mentioned were reported
Goretti et al. (42)	Consultation and involvement	Observational	Cohort study	Interviews	Patients were interviewed by clinicians to collect the patients and their family shared experiences (consultation) and suggestions to improve their pathway of care (involvement).	Patient experience Suggestions to improve the care pathway	The collection of experiences and suggestions led to three categories with a total of seven elements
Wickramasinghe et al. (38)	Consultation and involvement	Randomized	Crossover clinical trial	Questionnaire/survey	Patients were asked to filled out a structured questionnaire/survey at four specific stages to ascertain overall satisfaction with the technology solution (consultation) and allow for any recommendations moving forward (involvement).	Patient satisfaction Recommendations for alternate exercise	Five a priori themes were included, and thematic analysis uncovered two new themes

#### 3.6.1. Type of studies and their modalities

Six of seven studies have an observational design (31, 33, 36, 39, 40, 42), of which two are cohort studies (33, 42), two are cross-sectional studies (36, 39), one a cross-control study (40), and one a multiple case study (31). One of seven studies has a randomized design, which is a cross-over clinical trial (38).

#### 3.6.2. Data collection methods

Five of the seven studies used a questionnaire or survey (33, 36, 38–40), one in combination with interviews (40). In one study, interviews were the only data collection method used (42) and in one study interviews were combined with a multi-stakeholder panel (31).

#### 3.6.3. Outcomes of patient engagement

All seven studies reported the outcomes of both levels consultation and involvement. Three of seven studies reported three outcomes (31, 36, 40), three reported two outcomes (38, 39, 42) and one study reported four outcomes (33). At the level of consultation five studies reported patient experience as outcome of patient engagement (31, 33, 39, 40, 42), one study patient satisfaction (38), one study quality outcome measures and time and money saved (33), and one study treatment aspects and challenges for recovery (36). At the level of involvement two studies patient's advice as outcome of patient engagement (31, 36), two studies barriers and facilitators (39, 40), one study validation of concepts (31), one study preferences for further visits (33), one study added value of a healthcare monitor (40), one study suggestions to improve the care pathway (42), and one study recommendations for alternate exercise (38).

#### 3.6.4. Results reported

Six of seven studies show that the role of patients and their input is substantial used in the results and conclusion of the study (33, 36, 38–40, 42), and one study shows that it is used minimally (31). In the study which the results were minimally used there were just two patients that participated in a total of 48 participants (31). In the other six studies there were different results reported. One study mentioned patients' experiences, time and money saved and the preferred follow-up method (33), one study mentioned the high-quality criteria based on patients' answers (36), another study mentioned the patient and professional experiences and barriers and facilitators as equally important (39), another study mentioned the experiences and barriers and facilitators of two groups of patients (40), another study mentioned the experiences and suggestions which led to three categories with a total of seven elements to improve their pathway of care (42) and one study mentioned two new themes based on the thematic analysis with patients (38).

### 3.7. Involvement

Five studies addressed the involvement level (29, 30, 41, 47, 48). Further details can be found in Table 5 below.

**Table 5:** Involvement level of engagement.

**References**	**Level of engagement**	**Type of studies**	**Modalities**	**Data collection methods**	**Role of patients related to the level of engagement**	**Outcomes patient engagement**	**Results of patient engagement reported**
Anderson et al. (29)	Involvement	Observational	Cross-control study	Interviews	Patients viewed videos and commented on the overall approaches and specific discussion components	Patient preferences	The percentages of the patients preferring the different models are clearly mentioned
Slejko et al. (48)	Involvement	Observational	Case study	Questionnaire/survey	Patients were asked which value elements that were the most important to them in making decisions about treatment to manage their condition.	Domain and value element importance	Resulting in six subgroups of value elements important to a specific patient population
Hennink et al. (30)	Involvement	Observational	Cross-sectional study	Questionnaire/survey	Patients assessed the importance of outcomes and were invited to formulate their own outcomes	Relevance of outcomes	Showed confirmation by the patients of all six relevant outcomes
Fahner et al. (41)	Involvement	Observational	Cross-sectional study	Interviews Focus group	Patients were asked to elucidate the perspectives on contemplating the future	Underlying values Influence values for future decision-making	Categorized four main themes to envision the future
Najafabadi et al. (47)	Involvement	Observational	Cross-sectional study	Interviews	Patients discussed the whole meningioma care trajectory and for each part of the care trajectory the relevant themes. Patients were asked to identify issues as well as possible solutions for this issues.	Discussing care trajectory Identify issues Possible solutions	The grounded theory approach identified three issues and 16 possible solutions

#### 3.7.1. Type of studies and their modalities

All five studies have an observational design (29, 30, 41, 47, 48), of which three are cross-sectional studies (30, 41, 47), one cross-control study (29), and one case study (48).

#### 3.7.2. Data collection methods

Two studies used questionnaires (30, 48), two studies interviews (29, 47), and one study used interviews in combination with a focus group (41). The data collection methods in the five studies have led to different roles of patients in relation to the level “involvement” of patient engagement.

#### 3.7.3. Outcomes of patient engagement

All studies reported one outcome and there are different outcomes reported. One study focused on patient preferences (29), one on domain and value importance (48), one on the relevance of outcomes (30), one on the underlying values and influence of values for future decision-making (41), and one on discussing the care trajectory, the issues related to the care trajectory and the possible solutions for these issues (47).

#### 3.7.4. Results reported

All studies show that the role of patients and their input is substantial used in the results and conclusion of the study. One study mentioned the preferences of patients clearly (29), one study mentioned six different subgroups important to the specific patient population (48), another study provided confirmation by the patients of all six relevant outcomes (30), another categorized four main themes to envision the future (41), and one study identified, on the basis of a grounded theory approach, three issues and sixteen solutions for these issues (47).

## 4. Discussion

To the best of our knowledge, this is the first systematic review that investigates the communication between patients and healthcare providers at the organizational level of hospitals and throughout the full cycle of care in a VBHC setting. We found that it was most commonly interviews and questionnaires, that can be seen as examples of low-level engagement, that were deployed to engage patients in designing new care pathways and quality improvement projects. Higher-level engagement tools, such as focus groups, co-design experience, collaborative teams, advisory committees, and joint decision-making, are rarely used to improve healthcare in hospitals. This is remarkable in value-driven care approaches that claim to take patient-centeredness and creating patient value as the starting point.

This low level of patient engagement in VBHC is also illustrated by the roadmap for implementing VBHC that has recently been presented by an expert working group from nine large European University Hospitals (50). The roadmap does not pay any attention to patient engagement beyond the advice to develop patient-reported outcome measures (PROMs) and patient-reported experience measures (PREMs) to measure outcomes and experiences. Our conclusion that higher levels of patient engagement should be pursued is supported by the work of Berwick (3). Today, according to Berwick, we are in an era where there is great emphasis on mandatory measurements and a clash between professional autonomy and these tools for external accountability. Berwick emphasizes the importance of “hearing the voices of the people served” in what he envisions as a new era for medicine and healthcare. The expected benefits of this new era are reduced mutual distrust among by actors in the field, a greatly reduced administrative burden for all, and, by incorporating healthcare users of and their families in co-design activities, improved services.

VBHC research that focuses on the level of direct care demonstrates that patient-reported outcome measures are increasingly used in the consulting room to discuss treatment and outcome preferences between doctors and patients. PROMs could also be used to improve healthcare quality and result in higher levels of patient engagement such as shared decision-making (24–29, 31–33). However, we found that higher-level engagement is not yet current practice in VBHC initiatives at the organizational level. Furthermore, our review shows that the organization of the care process and improvements to care pathways are hardly influenced by patient engagement. Only one paper reported the implementation of an improved care process that was a result of patient engagement (32). The possibilities to improve care pathways by using high-level patient engagement strategies extend to experienced-based co-design, involving patient advocates in the organization of care and in influencing patient organizations (12, 16, 51–55). However, we also recognize the risk of tokenistic patient engagement (12, 16). Tokenistic engagement may demotivate patients to participate. To avoid this pitfall, the importance of “creating a receptive context” is stressed, along with open communication, honesty, and trust between doctors, patients, and other participants (12, 56).

Furthermore, the results of our systematic review at the organizational level show that, although low levels of patient engagement do inform healthcare providers about the values held by patients, once this input has been made by patients and their family members, they are no longer involved in improving healthcare services. Patients and families are rarely involved in collaborative thinking about ways to improve healthcare, even though the literature suggests that higher levels of patient engagement can increase the likelihood of improving care processes (12, 16, 52, 53, 57). To determine what is of value to patients, in other words what matters most to patients, patients have to be engaged in the development of healthcare services (52). To summarize, we believe that higher levels of patient engagement at the organizational level (e.g., involvement in redesigning care processes) can be of tremendous value when implementing VBHC.

### 4.1. Strengths and limitations

A strength of our study is that it focuses on organizational-level patient engagement in a VBHC setting, a field that to the best of our knowledge has not previously been addressed in a systematic review. This is a developing and relevant field because both VBHC and patient engagement are of growing importance in improving healthcare and in the ongoing shift from volume-driven to value-driven healthcare delivery. In addition, VBHC initially focuses on the needs of patients at the direct care level, whereby this systematic review shows that there are already 21 papers at the organizational level of patient engagement in a VBHC context.

There are five limitations in this study. First, we specifically included empirical research in the VBHC field that involved any form of patient engagement. By only including current research related to hospital care, we did not include primary care or chronic care for the elderly in nursing homes. The motivation for limiting ourselves to research involving hospitals was prompted by the fact that VBHC always aims to improve the full cycle of care, and so hospitals are always an element in this. Second, as a result of our 21 included articles, 19 papers were observational and two randomized. Due to this, there may be a limited level of evidence, however, this study shows that the observational articles contain a relatively large number of cohort and cross-control studies that are in the highest levels of observational studies (58). Third, due to the choice of MMAT as quality assessment tool, we did not analyze inconsistency and publication bias, which could be important items for assessing the quality of the studies. Fourth, we only included peer-reviewed publications, which may mean that we have overlooked relevant VBHC initiatives. Finally, the perspective of this study is limited to the definition of VBHC as introduced by Porter on patient and organizational level. Although Porter's definition may have evolved over time, especially in Europe, allocative and societal value play hardly a role in his definition.

## 5. Conclusion

This study included 21 articles, the majority of which were observational, resulting in a limited quality of evidence. Our main contribution is highlighting that extensive patient engagement, as a valuable approach to improving healthcare at the organizational level in a VBHC setting, is rarely used. Current engagement tools between care providers and patients rarely go beyond the communication level of interviews and questionnaires. While this form of communication may be of value to care providers seeking to improve healthcare, it ignores the possibilities of higher-level engagement such as co-design and collaboration. Higher-level engagement would provide an opportunity to improve healthcare and care pathways through co-production with the people being served. We would urge VBHC initiatives to embrace all levels of patient engagement to ensure that patient values find their way to the heart of these initiatives.

## Data availability statement

The original contributions presented in the study are included in the article/Supplementary material, further inquiries can be directed to the corresponding author.

## Author contributions

MV and WS: involved in the conceptualization of the study, development of the search strategy, screened all papers (first screener), formal analysis, and original draft and editing. MJ: involved in the resources, review, editing, and supervision. AF: involved in the conceptualization of the study, screened a subset of the articles (second screener), resources, review, editing, and supervision. KA: involved in the conceptualization of the study, development of the search strategy, screened a subset of the articles (second screener), resources, review, editing, and supervision. All authors approved the final version of the manuscript.
