# Supplementary material for: The immaturity of patient engagement in value-based healthcare—A systematic review
Source: Front Public Health. 2023 May 11;11:1144027. doi: 10.3389/fpubh.2023.1144027 (PMC10213745; doi:10.3389/fpubh.2023.1144027)
Supplement: Supplementary file 2 [file Data_Sheet_2.docx]

patient participation value based health care

| **Database searched** | **via** | **Years of coverage** | **Records** | **Records after duplicates removed** |
| --- | --- | --- | --- | --- |
| Embase | Embase.com | 1971 - Present | 901 | 214 |
| Medline ALL | Ovid | 1946 - Present | 786 | 782 |
| Web of Science Core Collection* | Web of Knowledge | 1975 - Present | 978 | 402 |
| Cochrane Central Register of Controlled Trials | Wiley | 1992 - Present | 50 | 4 |
| Google Scholar |  |  | 200 | 144 |
| **Total** | | | **2915** | **1546** |

*Science Citation Index Expanded (1975-present) ; Social Sciences Citation Index (1975-present) ; Arts & Humanities Citation Index (1975-present) ; Conference Proceedings Citation Index- Science (1990-present) ; Conference Proceedings Citation Index- Social Science & Humanities (1990-present) ; Emerging Sources Citation Index (2015-present)

**embase.com 900**

('value based care'/de OR 'value based medicine'/de OR (vbhc OR vb-hc OR ((value-based OR valuebased) NOT ((value-based OR valuebased) NEXT/2 (insuran* OR purchas* OR pric* OR reimburse* OR contract* OR payment* OR partnership*))) OR ((high-value OR value-driven) NEAR/3 (care OR healthcare)) OR (measuring NEAR/3 value NEAR/3 (care OR healthcare))):ab,ti) AND ('patient participation'/de OR 'patient engagement'/de OR 'shared decision making'/de OR 'patient decision making'/de OR 'patient-reported outcome'/de OR 'patient satisfaction'/de OR 'patient experience'/de OR 'doctor patient relationship'/de OR 'patient preference'/de OR 'patient education'/de OR 'patient advocacy'/de OR (((patient* OR user*) NEAR/3 (participat* OR involve* OR engage* OR voice* OR inclusion* OR satisfaction* OR experience* OR relation* OR preference* OR decision* OR education* OR council* OR consult* OR advocac* OR perspective*)) OR (shared NEAR/3 decision*) OR co-design* OR codesign* OR (patient* NEXT/1 (report*)) OR prom OR proms):ab,ti) NOT [conference abstract]/lim AND [english]/lim

**Medline ALL**

((vbhc OR vb-hc OR ((value-based OR valuebased) NOT ((value-based OR valuebased) ADJ2 (insuran* OR purchas* OR pric* OR reimburse* OR contract* OR payment* OR partnership*))) OR ((high-value OR value-driven) ADJ3 (care OR healthcare)) OR (measuring ADJ3 value ADJ3 (care OR healthcare))).ab,ti.) AND (Patient Participation/ OR Decision Making, Shared/ OR c/ OR Patient Satisfaction/ OR Physician-Patient Relations/ OR Patient Preference/ OR Patient Advocacy/ OR (((patient* OR user*) ADJ3 (participat* OR involve* OR engage* OR voice* OR inclusion* OR satisfaction* OR experience* OR relation* OR preference* OR decision* OR education* OR council* OR consult* OR advocac* OR perspective*)) OR (shared ADJ3 decision*) OR co-design* OR codesign* OR (patient* ADJ (report*)) OR prom OR proms).ab,ti.) NOT (conference abstract) AND english.la.

**Web of Science Core Collection***

TS=((vbhc OR vb-hc OR ((value-based OR valuebased) NOT ((value-based OR valuebased) NEAR/2 (insuran* OR purchas* OR pric* OR reimburse* OR contract* OR payment* OR partnership*))) OR ((high-value OR value-driven) NEAR/2 (care OR healthcare)) OR (measuring NEAR/2 value NEAR/2 (care OR healthcare))) AND (((patient* OR user*) NEAR/2 (participat* OR involve* OR engage* OR voice* OR inclusion* OR satisfaction* OR experience* OR relation* OR preference* OR decision* OR education* OR council* OR consult* OR advocac* OR perspective*)) OR (shared NEAR/2 decision*) OR co-design* OR codesign* OR (patient* NEAR/1 (report*)) OR prom OR proms)) AND DT=(Article OR Review OR Letter OR Early Access) AND LA=(English)

**Cochrane Central Register of Controlled Trials**

((vbhc OR vb NEXT hc OR ((value NEXT based OR valuebased) NOT ((value NEXT based OR valuebased) NEXT/2 (insuran* OR purchas* OR pric* OR reimburse* OR contract* OR payment* OR partnership*))) OR ((high NEXT value OR value NEXT driven) NEAR/3 (care OR healthcare)) OR (measuring NEAR/3 value NEAR/3 (care OR healthcare))):ab,ti) AND ((((patient* OR user*) NEAR/3 (participat* OR involve* OR engage* OR voice* OR inclusion* OR satisfaction* OR experience* OR relation* OR preference* OR decision* OR education* OR council* OR consult* OR advocac* OR perspective*)) OR (shared NEAR/3 decision*) OR co NEXT design* OR codesign* OR (patient* NEXT/1 (report*)) OR prom OR proms):ab,ti) NOT (conference abstract)

**Google Scholar**

"value based"|valuebased "patient|user participation|involvement|engagement|voice|inclusion|satisfaction|experience|relation|preference|decision|education|council|consultation|advocacy|perspective"|"shared decision"|"co-design"|codesign|"patient reported"
