# Supplementary material for: The immaturity of patient engagement in value-based healthcare—A systematic review
Source: Front Public Health. 2023 May 11;11:1144027. doi: 10.3389/fpubh.2023.1144027 (PMC10213745; doi:10.3389/fpubh.2023.1144027)
Supplement: Supplementary file 3 [file Data_Sheet_3.docx]

Quality appraisal using the Mixed Method Appraisal Tool (MMAT)

# How MMAT has been used to appraise quality

- All included studies have been subjected to the two generic screening items (see below).
- Qualitative and quantitative studies have been subjected to their unique category of screening items (see below).
- Mixed methods studies have been subjected to all three screening categories (qualitative, quantitative and mixed method) (see below).
- Response options were: Yes, No and Can’t tell.

# Response to score conversion

As suggested in the MMAT 2018 guideline (Hong et al, 2018), ‘Yes’ responses have been scored ‘1’ and ‘No’ and ‘Can’t tell’ responses have been scored ‘0’. Each category can receive a maximum score of 5. The score ‘5’ means that 100% of the quality criteria are met. Respectively, a score of 0 means that none of the quality criteria were met. For mixed method studies the category with the lowest scoring assessment was used, because studies cannot exceed the quality of its weakest component.

MMAT does not provide cut off values to characterize ‘low’, ‘medium’ and ‘high’ quality studies. Authors are free in this choice as long as the chosen cutoff values are transparent. In this review three categories (low, medium, and high quality) were used, representing studies with scores 0-2, 3 and 4-5 respectively.

# Screening items (Hong et al, 2018)

## Screening (all studies)

S1. Are there clear research questions?

S2. Do the collected data allow to address the research questions?

## Qualitative

1.1. Is the qualitative approach appropriate to answer the research question?

1.2. Are the qualitative data collection methods adequate to address the research question?

1.3. Are the findings adequately derived from the data?

1.4. Is the interpretation of results sufficiently substantiated by data?

1.5. Is there coherence between qualitative data sources, collection, analysis and interpretation?

## Quantitative descriptive

4.1. Is the sampling strategy relevant to address the research question?

4.2. Is the sample representative of the target population?

4.3. Are the measurements appropriate?

4.4. Is the risk of nonresponse bias low?

4.5. Is the statistical analysis appropriate to answer the research question?

## Mixed methods

5.1. Is there an adequate rationale for using a mixed methods design to address the research question?

5.2. Are the different components of the study effectively integrated to answer the research question?

5.3. Are the outputs of the integration of qualitative and quantitative components adequately interpreted?

5.4. Are divergences and inconsistencies between quantitative and qualitative results adequately addressed?

5.5. Do the different components of the study adhere to the quality criteria of each tradition of the methods involved?

# Results

All included studies rated ‘Yes’ to the two screening questions, indicating that MMAT can be used as a tool for further assessment. These screening questions were left out in the table below.

| Study | Criteria from the Mixed Method Appraisal Tool (MMAT 2018) | | | | | | | | | | | | | | | Score | Quality | Explanations |
| --- | --- | --- | --- | --- | --- | --- | --- | --- | --- | --- | --- | --- | --- | --- | --- | --- | --- | --- |
|  | 1.1 | 1.2 | 1.3 | 1.4 | 1.5 | 4.1 | 4.2 | 4.3 | 4.4 | 4.5 | 5.1 | 5.2 | 5.3 | 5.4 | 5.5 |  |  |  |
| Qualitative designs | | | | | | | | | | | | | | | | | |  |
| (Fahner et al., 2020) | 1 | 1 | 1 | 1 | 1 |  |  |  |  |  |  |  |  |  |  | 5 | High | Solid method, resulting in motivated participants |
| (Anderson et al., 2014) | 1 | 1 | 0 | 1 | 0 |  |  |  |  |  |  |  |  |  |  | 3 | Medium | Clearly described method section. The research question had two aims, where one of the two was not explicitly discussed in the results |
| (Eppler et al., 2019) | 1 | 1 | 1 | 1 | 1 |  |  |  |  |  |  |  |  |  |  | 5 | High | Clear research question, method used and outcomes |
| (Slejko et al., 2021) | 1 | 1 | 1 | 0 | 1 |  |  |  |  |  |  |  |  |  |  | 4 | High | To understand patient value elements, many value elements were derived from a relatively small number of patients |
| (Kaplan et al., 2014) | 1 | 1 | 1 | 1 | 1 |  |  |  |  |  |  |  |  |  |  | 5 | High | Clear and transparent description of the study, met the quality criteria, the representative sample size could be questioned |
| Quantitative designs | | | | | | | | | | | | | | | | | |  |
| (Hennink et al., 2013) |  |  |  |  |  | 1 | 1 | 1 | 0 | 1 |  |  |  |  |  | 4 | High | Strong outcomes, response 59% from the selected patient group |
| (Li et al., 2017) |  |  |  |  |  | 1 | 1 | 1 | 1 | 1 |  |  |  |  |  | 5 | High | Clear research question, method used and outcomes |
| (Bernstein et al., 2019) |  |  |  |  |  | 1 | 0 | 1 | 0 | 1 |  |  |  |  |  | 3 | Medium, | Surgeons elected participants. The majority of participants is female, white and have high self-rated mental and overall health. Half of patients visiting the clinic had college degrees |
| (Ahluwalia et al., 2021) |  |  |  |  |  | 1 | 0 | 1 | 0 | 1 |  |  |  |  |  | 3 | Medium | Too small sample size divided over two groups that were not equal compared to the target population. The quantitative analysis was carried out minimally, which can lead to a distorted view of the results |
| (Kasalak et al., 2022) |  |  |  |  |  | 1 | 1 | 1 | 0 | 1 |  |  |  |  |  | 4 | High | There may be a selection bias due to the percentage of completed questionnaires. Large percentage difference in size of randomized groups and significant difference between both groups with regard to referring speciality |
| (Van Veghel et al., 2020) |  |  |  |  |  | 1 | 1 | 1 | 0 | 1 |  |  |  |  |  | 4 | High | There may be an information bias due to retrospective research, in which patient satisfaction was measured over more than three years ago |
| (Young et al., 2020) |  |  |  |  |  | 1 | 1 | 0 | 0 | 1 |  |  |  |  |  | 3 | Medium | Statistical analysis was not described in detail. Due to this, in combination with the retrospective research, there is an increased risk of information bias. Unequal ratio between the two groups compared in the study |
| (Coppess et al., 2018) |  |  |  |  |  | 0 | 0 | 1 | 1 | 1 |  |  |  |  |  | 3 | Medium | Sampling strategy and target population were not described |
| (Rosseel et al., 2019) |  |  |  |  |  | 1 | 1 | 1 | 1 | 1 |  |  |  |  |  | 5 | High | Representative sample and both groups were well presented. Clear description of the statistical analysis that fitted well with the research question |
| Mixed Method designs | | | | | | | | | | | | | | | | | |  |
| (Pennucci et al., 2019) | 1 | 0 | 1 | 1 | 1 | 1 | 1 | 1 | 1 | 1 | 0 | 1 | 1 | 1 | 0 | 3 | Medium | Patients were engaged in collecting quantitative data, professionals interpreted outcomes in workshops. The questionnaires were more clearly elaborated and would better meet the research question |
| (Dronkers et al., 2020) | 1 | 1 | 1 | 1 | 1 | 1 | 1 | 1 | 1 | 1 | 1 | 1 | 1 | 1 | 1 | 5 | High | Clear structure with motivated use of mixed methods approach and outcomes formulated as lessons learned |
| (Goretti et al., 2020) | 1 | 1 | 1 | 1 | 1 | 1 | 1 | 1 | 1 | 0 | 1 | 1 | 1 | 0 | 1 | 4 | High | Clear ambition to employ VBHC principles, methods rationale were not entirely clear |
| (Najafabadi et al., 2020) | 1 | 1 | 1 | 1 | 1 | 1 | 1 | 1 | 1 | 1 | 1 | 1 | 1 | 1 | 1 | 5 | High | Clear explanation of the sample strategy. Respondents were used at several moments in the research, which in total led to a representative sample size. Clear integration between qualitative and quantitative data |
| (Wickramasinghe et al., 2019) | 1 | 1 | 1 | 1 | 1 | 1 | 0 | 0 | 1 | 1 | 1 | 1 | 0 | 1 | 1 | 3 | Medium | Small sample size presented in the study due to ethical restrictions. Quantitative data was barely described. Little integration between qualitative and quantitative data |
| (Van Citters et al., 2014) | 1 | 1 | 1 | 1 | 1 | 1 | 0 | 1 | 0 | 1 | 1 | 1 | 0 | 1 | 1 | 3 | Medium | Small sample size that were not representative for the target population. Due to the small sample size and patients that were not included in the multi-stakeholder panel for developing a new care pathway, there is an increased risk of bias |
| (Depla et al., 2020) | 1 | 0 | 1 | 1 | 1 | 1 | 1 | 1 | 1 | 1 | 1 | 1 | 1 | 1 | 1 | 4 | High | The qualitative data was collected via an open-ended survey, but focus groups or interviews would be more suitable for this |

# Results summary

| Study Design | Number of studies (total) | Number of studies per MMAT quality appraisal score category |
| --- | --- | --- |
| Qualitative | 5 | 4 High |
|  |  | 1 Medium |
|  |  | 0 Low |
| Quantitative | 9 | 5 High |
|  |  | 4 Medium |
|  |  | 0 Low |
| Mixed Method | 7 | 4 High |
|  |  | 3 Medium |
|  |  | 0 low |
